# The role of Clec11a in bone construction and remodeling

**DOI:** 10.3389/fendo.2024.1429567

**Published:** 2024-08-12

**Authors:** Ke Xu, Rui-qi Huang, Ruiming Wen, Yao Yang, Yang Cheng, Bo Chang

**Affiliations:** ^1^ School of Sports Health, Shenyang Sport University, Shenyang, Liaoning, China; ^2^ Laboratory Management Center, Shenyang Sport University, Shenyang, Liaoning, China; ^3^ School of Sport Science, Zhuhai College of Science and Technology, Zhuhai, Guangdong, China

**Keywords:** Clec11a, skeletal system, bone construction, bone remodeling, exercise

## Abstract

Bone is a dynamically active tissue whose health status is closely related to its construction and remodeling, and imbalances in bone homeostasis lead to a wide range of bone diseases. The sulfated glycoprotein C-type lectin structural domain family 11 member A (Clec11a) is a key factor in bone mass regulation that significantly promotes the osteogenic differentiation of bone marrow mesenchymal stem cells and osteoblasts and stimulates chondrocyte proliferation, thereby promoting longitudinal bone growth. More importantly, Clec11a has high therapeutic potential for treating various bone diseases and can enhance the therapeutic effects of the parathyroid hormone against osteoporosis. Clec11a is also involved in the stress/adaptive response of bone to exercise via mechanical stimulation of the cation channel Pieoz1. Clec11a plays an important role in promoting bone health and preventing bone disease and may represent a new target and novel drug for bone disease treatment. Therefore, this review aims to explore the role and possible mechanisms of Clec11a in the skeletal system, evaluate its value as a potential therapeutic target against bone diseases, and provide new ideas and strategies for basic research on Clec11a and preventing and treating bone disease.

## 1 Introduction

During the period from early childhood into adulthood, bone tissue continuously undergoes dynamic changes of construction and remodeling to accommodate bone growth and development in early childhood and maintain its function in adulthood (1, 2). Osteocytes, osteoclasts (OCs), osteoblasts (OBs) and chondrocytes interact through a sophisticated network of molecular signaling to synergistically regulate bone construction and remodeling. This process involves the secretion and delivery of a variety of cytokines, forming a complex yet ordered regulatory mechanism. Once this molecular signaling network is imbalanced, bone construction and remodeling become dysregulated, which may result in the development of various bone diseases such as osteoporosis (OP) and osteosclerosis (3, 4). Therefore, precise regulation of the signaling network is essential for maintaining bone health and preventing bone diseases.

**Figure 1:**
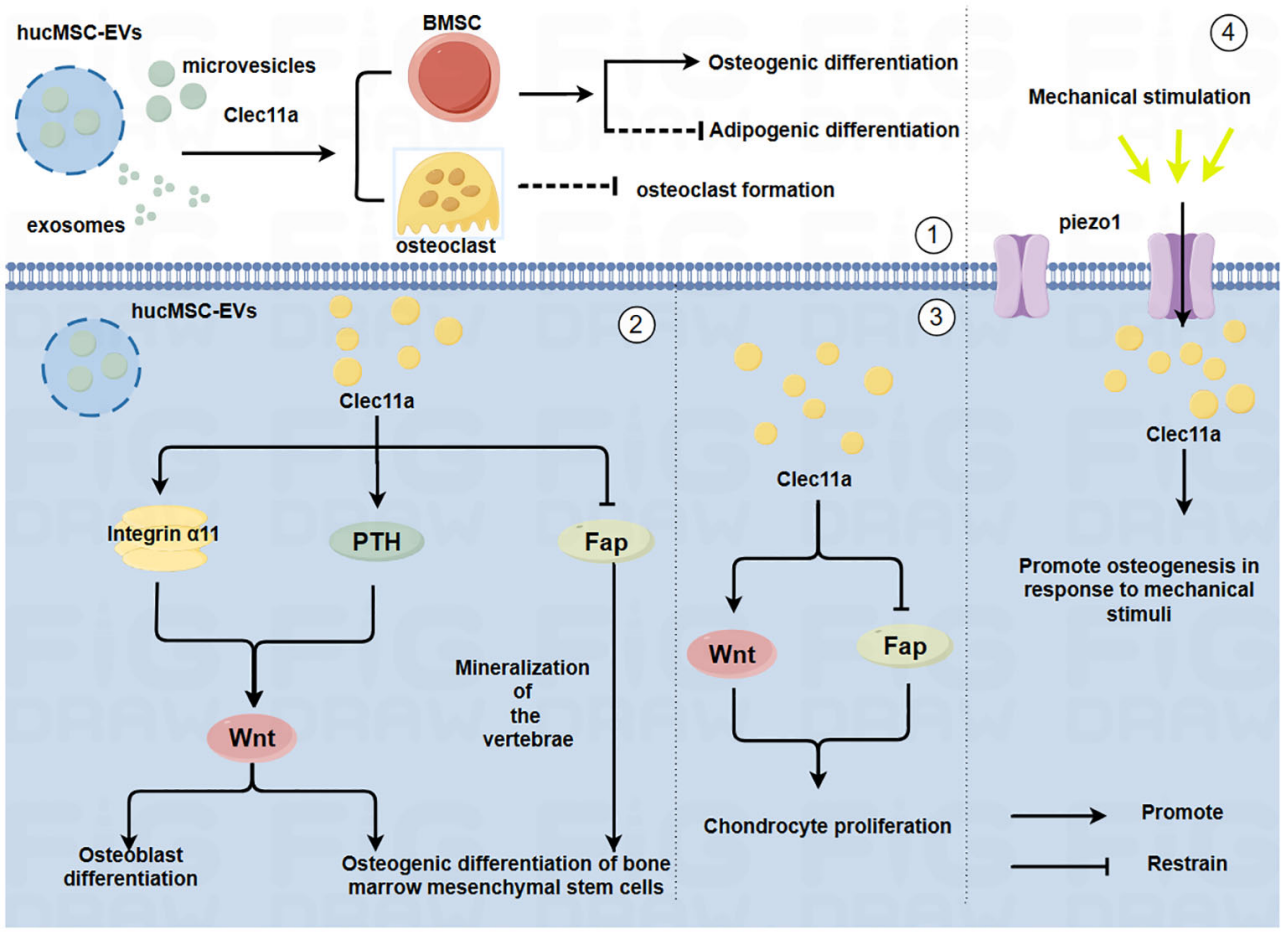
Mechanism by which Clec11a regulates bone cells and its relationship with mechanical forces(By Figdraw.) ①HucMSC-EVs may promote the osteogenic differentiation of BMSCs, inhibit the adipogenic differentiation of BMSCs and inhibit the generation of OC through Clec11a. ②Clec11a promotes the osteogenic differentiation of BMSCs and OB by binding to integrin α11 in response to Wnt signaling. Clec11a promotes the osteogenic differentiation of BMSCs through PTH activating Wnt signaling. Clec11a promoted vertebral bone mineralization and osteogenic differentiation of BMSCs by inhibiting FAP. ③Clec11a activates Wnt signaling to promote the proliferation of chondrocytes. Clec11a promotes chondrocyte proliferation by inhibiting Fap. ④Clec11a promotes osteogenesis through Piezo1 channel in response to mechanical stimulation.

Exploring the cytokines that affect bone metabolism and mechanisms that induce bone metabolism imbalance will provide an important theoretical basis for the prevention and treatment of bone diseases. Numerous cytokines are involved in the regulation of the skeletal system, among them, C-type lectin structural domain family 11 member A (Clec11a) is encoded as a member of the C-type lectin protein superfamily IX, which is also known as C-type lectin superfamily member 3 (CLECSF3) (5). Previous research primarily focusing on the role of Clec11a in a variety of hematopoietic and myeloid cells revealed its role as a stem cell growth factor (SCGF) (6). In 2016, Yue et al. found that Clec11a contributes to adult bone health by promoting the osteogenic differentiation of bone marrow mesenchymal stem cells (BMSCs); thus, Clec11a was subsequently identified as a bone lectin (7). Over time, the role of Clec11a in bone development and health has become increasingly clear. Studies have shown that it plays a role in inducing the osteogenic differentiation of BMSCs and promoting the proliferation of chondrocytes (8, 9). However, there is still some controversy about the specific role of Clec11a in the regulation of OC; some studies have reported that it has no significant effect on bone resorption, whereas other studies have reported that the absence of Clec11a promotes the formation of mature OC (7, 8). Moreover, in a transgenic mouse model using a Clec11a-driven fluorescent reporter to label osteoblast lineage cells, the researchers found that modest mechanical stimulation promoted an increase in overall bone mineral density and cortical thickness in mice and led to a significant upregulation of the number of Clec11a-positive cells surrounding the arterioles (10). This finding suggest there is a potential relationship between Clec11a and exercise and that exercise plays a role in promoting bone health. In summary, Clec11a may be closely associated with the skeletal system and has great potential for the treatment of skeletal system diseases (**Figure 1**).

Studies have demonstrated the role of the C-type lectin protein superfamily in the skeletal system (11); however, its functions and mechanisms in the skeletal system have not been summarized in detail. Therefore, this review focused on the role and mechanism of Clec11a in regulating the skeletal system and mediating bone health during exercise, based on information from relevant literature, and highlights the potential pharmacological value of Clec11a for the treatment of bone diseases.

## 2 Clec11a overview

C-type lectins are a superfamily of proteins that bind specifically to carbohydrates. As early as 1988, Drickamer et al. classified Ca2+ -dependent lectins with structurally similar glycoprotein receptors as C-type lectins (12). Clec11a, a member of the C-type lectin protein superfamily IX, was first identified in 1998 (13) and has been characterized as a secreted glycoprotein. Structural analysis showed that Clec11a includes an N-terminal RGD (Arg-Gly-Asp) triplet region, an α-helical leucine zipper dimerization domain consisting of six heptamers in the middle, and a C-terminal C-type lectin structural domain (13). The product encoded by the human Clec11a gene has two forms, SCGFα and SCGFβ: SCGFα is a full-length isoform with a molecular weight of 47 kDa, and SCGFβ is a shortened isoform with a molecular weight of 40 kDa that lacks the 78 aa lectin structural domain at the C-terminus (5, 13, 14).

Multiple sequence comparisons have shown that Clec11a homologous gene sequences are highly similar in humans, dogs, rats, mice, bovines, and primates (15), suggesting the potential conservation of the function of Clec11a across different species. Clec11a is distributed in a variety of tissues and especially highly expressed in BMSCs, chondrocytes, OB-like cells (6, 15, 16), indicating that Clec11a might be directly involved in bone-related physiological processes.*In situ* hybridization experiments on mouse fetuses and their tibias have shown that Clec11a is specifically expressed around skeletal tissues, and the gene expression of mouse Clec11a can also be detected in bone marrow cells, proliferating chondrocytes, primary ossification centers, perichondrium, and periosteum cells (6, 17). Based on the above, it is hypothesized that Clec11a is widely expressed in bone-related cells and bone tissues and is highly likely to play a crucial role in physiological processes such as bone growth, development, and repair.

## 3 Clec11a is involved in the regulation of bone construction and remodeling

Bone cells act as sensors and mediators to sense and transduce mechanical signals, coordinate bone homeostasis, and promote bone construction and remodeling, mainly including BMSCs, OB, OC, chondrocytes, and osteocytes, which exert unique coupling roles to maintain the balanced activity of each cell group and ensure balanced outcomes at the site of bone construction and remodeling (18, 19).

Bone construction refers to the formation of new bone tissue, including bone growth and development and changes in bone morphology. In bony vertebrates, bones develop mainly through two modes: endochondral osteogenesis and intramembranous osteogenesis (20). During intrachondral osteogenesis, mesenchymal precursor cells aggregate and differentiate into chondrocytes, thereby secreting cartilage matrix and forming hyaline cartilage rudiments. Subsequently, bone progenitor cells in the inner layer of the perichondrium of these cartilage rudiments differentiate into OB and form the bony collar. Thereafter, chondrocytes in the mid-cartilage segment become hypertrophic, leading to matrix mineralization and chondrocyte apoptosis. Then, OCs break down and absorb the calcified cartilage matrix, while OBs generate new bone on the surface of the residual cartilage matrix (21, 22). Intramembranous osteogenesis involves the direct differentiation of mesenchymal stem cells into OBs, which form a mineralized matrix in the ossification center and gradually expand. Bone remodeling refers to the repair and adjustment of bone tissue, which also results from a series of cellular events, mainly mediated by OB, OC, and osteocytes. Among them, OBs derived from BMSCs regulate bone formation (23) to maintain bone toughness and stiffness (24, 25). Bone resorption is regulated by OCs, which dissolve and absorb old bone matrix formed by calcium accumulation (26). Osteocytes form a large number of synapses with OB, OC, and other cells to promote the metabolism of mature bone (27).

**Figure 2:**
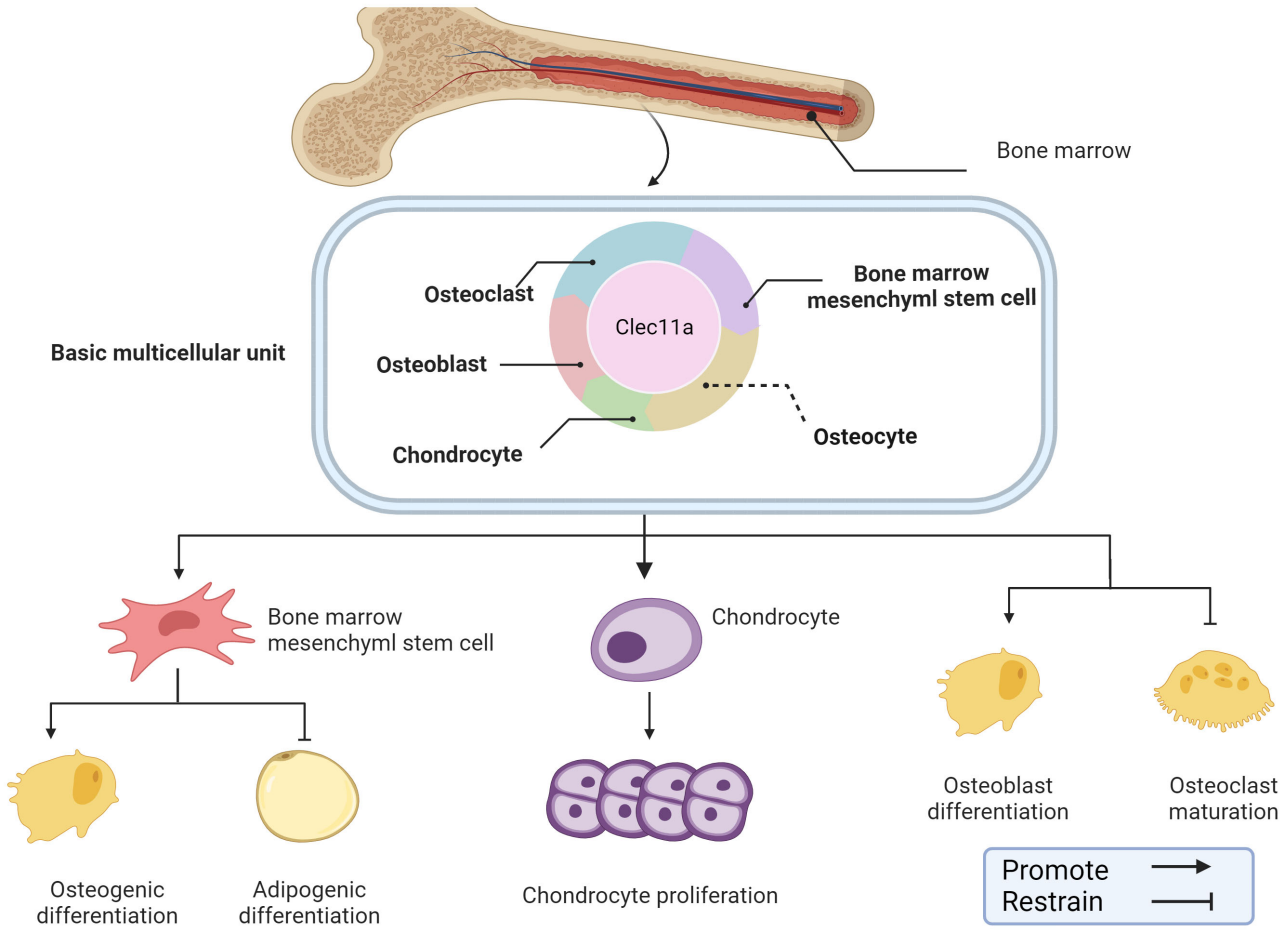
Role of Clec11a in bone construction and remodeling. Clec11a plays a regulatory role in a variety of cells in the skeletal system, including BMSCs, OB, OC, chondrocytes, etc. Clec11a can regulate BMSCs differentiation tendency, promote chondrocyte proliferation, promote OB differentiation and inhibit OC production.

Bone construction is predominant during the growth phase when the bone tissue is focused on growth and development (28). However, in adulthood, bone remodeling becomes dominant to maintain its strength and structure when receiving stress and load (29). In general, bone construction provides the foundation for bone remodeling, which is responsible for maintaining and optimizing the constructed bone, working synergistically to ensure that the bones can adapt to the changing needs of the body and environmental influences. Clec11a has been identified as an emerging regulator of the skeletal system that plays a regulatory role in various cells within the skeletal system, and is likely a key component in bone construction and remodeling. For example, Clec11a may promote osteogenic differentiation of BMSCs, inhibit the formation of mature OCs, and promote chondrocyte proliferation (**Figure 2**).

### 3.1 Clec11a regulates BMSCs

In 2016, Yue et al. found for the first time that Clec11a promotes the regulation of skeletal bone mass in adults (7). A combination of androgenic haploid embryonic stem cells (AG-haESCs) and regularly interspaced palindromic repeat sequence/CRISPR-associated protein 9 (CRISPR-Cas9) skeletal analyses identified Clec11a as a key gene for healthy bone development (30). Clec11a knockout mice displayed normal hematopoiesis at early developmental stages but had significantly reduced trabecular volume in their limb bones and vertebrae (7). Moreover, Clec11a knockout mice also showed accelerated bone loss, decreased bone strength, and delayed fracture healing during aging (7). *In vitro*, Clec11a-deficientBMSCs also showed impaired osteogenic differentiation but did not show changes in lipogenic and chondrogenic differentiation. Consistent with this finding, subcutaneous injection of recombinant Clec11a increased bone mass and osteogenic differentiation of BMSCs in osteoporotic mice (7). Moreover, Clec11a regulates BMSCs in a dose-dependent manner, suggesting that Clec11a may promote BMSCs bone formation and therefore regulate bone mass (31). The above results suggest that Clec11a promotes the differentiation of BMSCs into mature OBs to maintain adult bone health and plays a crucial role in maintaining adult bone health; however, the mechanism of action is not well understood and requires further research.

#### 3.1.1 BMSC-derived extracellular vesicles regulate bone metabolism through Clec11a

Extracellular vesicles (EVs) are membrane-encapsulated vesicles released by different types of cells, and they are mainly divided into three categories: exosomes, microvesicles, and apoptotic bodies. EVs are unique in their ability to exhibit similar effects as their parental cells (32–36). Studies have shown that BMSC-derived EVs (BMSC-EVs) can improve bone formation in OP through the Wnt/β-catenin signaling pathway (37), suggesting that BMSCs and BMSC-EVs have similar roles.

Recent reports have shown that tail vein injection of human umbilical cord mesenchymal stem cell-derived extracellular vesicles (hucMSC-EVs) in OP mice prevents bone loss and maintains bone strength by enhancing osteogenic differentiation and decreasing bone marrow fat accumulation (8). Proteomic analysis revealed that Clec11a was highly enriched in hucMSC-EVs and showed that silencing Clec11a in hucMSC-EVs significantly reduced the osteogenic differentiation of BMSCs and significantly downregulated the mRNA of the osteogenic differentiation markers Runx2, Sp7, Col1a1, Bglap, and Dmp1 compared with that of the hucMSC-EV-treated group (8). This result reveals a high potential that Clec11a is involved in regulating bone metabolism, indicating that hucMSC-EVs may regulate BMSCs osteogenic differentiation through Clec11a. Whether Clec11a regulates osteogenic activity through other parental cells of BMSCs remains unknown. However, the cited study presented certain limitations, and the silencing of Clec11a did not completely block the beneficial effects of hucMSC-EVs on bone metabolism *in vitro* because hucMSC-EVs were also present in other osteoprotegerins. Therefore, further studies are needed to investigate the mechanism of Clec11a in hucMSC-EVs and reveal the potential associations between Clec11a and other BMSCs parental cells.

#### 3.1.2 Activation of Clec11a target genes promotes BMSCs bone formation through Wnt signaling

Integrin alpha 11 (encoding Itga11) is a physiologically important receptor protein for Clec11a that is mainly expressed in leptin receptor (LepR) cells in BMSCs, as well as OBs, and can bind Clec11a with nanomolar affinity (38). Integrin signaling can promote Wnt pathway activation through integrin-linked kinase (ILK)-mediated GSK3 phosphorylation and β-linker protein nuclear translocation (39, 40), suggesting that integrin α11 may be closely related to bones physiology.

Recent reports have shown that recombinant Clec11a promotes the activation of Wnt signaling in BMSCs (38). Blockade of the Wnt signaling pathway in BMSCs using the Wnt signaling pathway transduction inhibitor IWR-1-endo blocked the osteogenic effects of Clec11a (38). Clec11a activates the Wnt signaling pathway by binding to its receptor integrin α11 to promote osteogenesis (38). The regulation of bone metabolism by Clec11a also involves other integrin receptors, such as integrin α10, which is most closely related to integrin α11; the former is widely expressed in chondrocytes and also has a nanomolar affinity for Clec11a (41, 42). The effects of Clec11a on bone cells with other integrin receptors and the interaction between the two should be further explored in subsequent studies. In addition to acting as receptors, integrins also serve as sensors in response to mechanical stimuli (43). Skeletal stem cells from the developing jawbone can also promote osteogenesis in response to mechanical forces by activating FAK, a molecule fundamental to the integrin-dependent signaling pathway (44). Whether Clec11a can promote bone health by binding to integrin α11 in response to mechanical stimuli is an extremely interesting question that should be further explored and validated by large-scale and multicenter cutting-edge studies.

Clec11a promotes vertebral mineralization in zebrafish by inhibiting fibroblast activation proteins (Fap or Seprase) (45). The use of a highly selective inhibitor of Fap significantly upregulates typical Wnt pathway-related genes (45), suggesting that Clec11a promotes bone formation in zebrafish through the Fap-regulated Wnt signaling pathway. Although this study mainly investigated the interaction between Clec11a and Fap in zebrafish and may therefore provide insights on the role of Clec11a in bone. Differences in the effect of Clec11a between species must also be considered, such as the effect of Clec11a on the phenotype of mice, as well as the expression pattern, developmental stage, and other factors.

In addition, recent studies have found that supplementation with 10 ng/ml of Clec11a significantly increases ALP activity and enhances the mineralization capacity of human dental pulp cells during the process of induced differentiation of human dental pulp cells (46). It is worth mentioning that ERK, JNK, and AKT signaling were activated just 5 minutes after Clec11a stimulation. Pretreatment of cells with ERK, JNK, and AKT inhibitors not only led to a significant down-regulation of ALP activity and mineralization levels in the inhibitor pretreatment group but also led to a sharp decrease in the expression levels of osteogenesis-related genes and proteins (46). It is well known that the normal regulation of ERK, JNK, and AKT signaling plays a crucial role in maintaining the normal metabolism and structural integrity of bones (47, 48). These results suggest that Clec11a may play a role in the regulation of typical bone metabolic signaling pathways, and the precise potential signal transduction mechanism of Clec11a in bone and the exact link between these signaling pathways can be further explored in the future.

**Figure 3:**
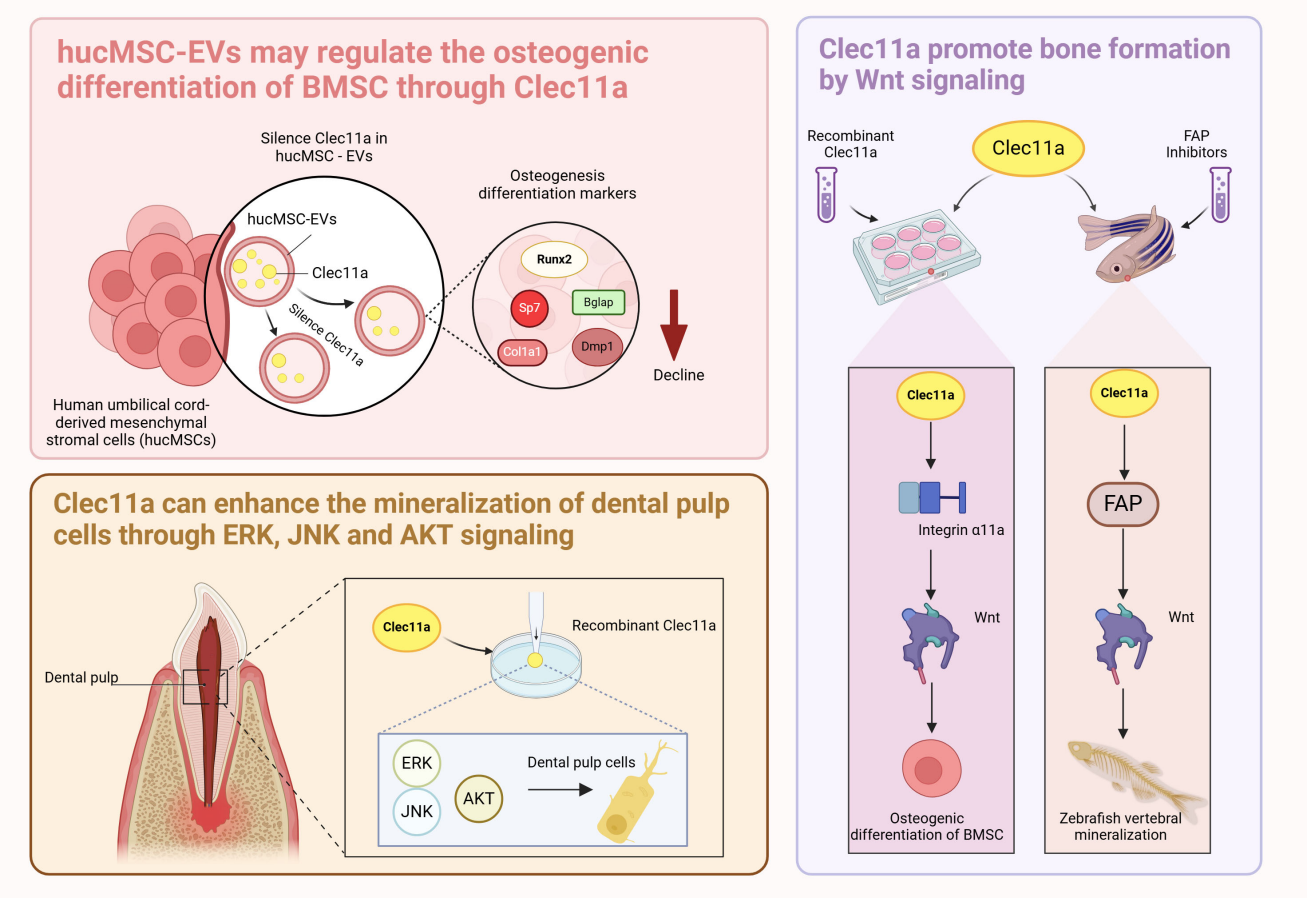
Role of Clec11a in BMSCs and dental pulp cells.

In summary, the mechanism of Clec11a regulating BMSCs is complex and extensive. Clec11a can mediate hucMSC-Evs and activate the target genes integrin α11 and Fap to affect Wnt signaling and promote the osteogenic differentiation of BMSCs. The molecular mechanisms of the relationship between Clec11a and hucMSC-Evs, integrin α11, and Wnt signaling can be further explored to provide a stronger theoretical basis for the regulation of bone health by Clec11a (**Figure 3**). In addition, the current question of whether Clec11a regulates bone metabolism through other mechanisms is worthy of further exploration. A deeper investigation into the mechanism of Clec11a regulation of bone metabolism can provide a theoretical basis for the prevention and treatment of bone diseases and the development of new clinical drugs.

### 3.2 Clec11a regulates chondrocyte proliferation

During the process of bone construction and remodeling, the proliferation and differentiation of chondrocytes are of vital importance. The proliferation of chondrocytes provides the material basis and spatial conditions for the formation of long bones and is one of the key links in the process of long bone formation. In juvenile mammals, chondrocytes in the growth plate can achieve longitudinal bone growth through columnar proliferation (49). However, chondrocyte proliferation is a complex developmental process influenced by various factors, such as hormones and signaling pathways (50, 51).

It has been reported that the knockout of Clec11a has no effect on the formation or growth of mouse bones before 14 days of birth, but the femur length of mice was shortened by 3~4% at four weeks of age, and the length of femur and vertebrae (3~4%) was shortened at 8 weeks of age, and the volume, number and thickness of trabecular bone were significantly reduced (9). Interestingly, no difference in the percentage of growth plate chondrocytes was observed in the 4-, 14-, and 8-week-old Clec11a knockout mice, whereas those in the 4-week-old Clec11a knockout mice were significantly reduced (9), suggesting that this reduced osteogenesis and bone elongation in the 4-week-old Clec11a knockout mice may be due to decreased chondrocyte proliferation. In stark contrast, intraperitoneal injection of 200 μg/kg Clec11a daily to 2-week-old wild mice for 6 weeks not only increased the femur length of juvenile mice (3~4%) but also increased the number of chondrocytes in 4-week-old mice. These reports indicate Clec11a may achieve longitudinal bone growth by promoting chondrocyte proliferation in juvenile mice, especially 4-week-old mice, and is a positive regulator of bone elongation in juvenile mice (9).

Notably, Zhang et al. utilized the Prx1 - cre technique to establish a mouse model with the Itga11 gene knocked out in mesenchymal stem cells, with the aim of investigating the influence of Itga11 on long bone formation and chondrocyte proliferation (9). The results indicated that at days 4 and 14, the formation and growth of limb bones were not significantly affected (9). However, at 4 weeks of age, the chondrocytes in the growth plate of mice decreased significantly, and the length of the femur shortened. Based on this, the authors employed the Aggrecan CreER technique to conditionally knockout the Itga11 gene in chondrocytes. The results showed that at 4 and 8 weeks of age, the femurs and vertebrae of Acan^CreER^;Itga11^fl/fl^ mice were shortened (9). At 8 weeks of age, the femurs and vertebrae of Acan^CreER^;Itga11^fl/fl^ mice were significantly shortened by 4% to 6%. The above suggests that Itga11 acts in a cell-autonomous manner in chondrocytes to promote proliferation and achieve longitudinal bone growth (9). This is consistent with observations in Clec11A-deficient mice. Further studies showed that Clec11a/integrin α11 both appear to promote chondrocyte proliferation for longitudinal bone growth through activation of the Wnt pathway (9). As previously mentioned, activation of Wnt signaling by Clec11a and its receptor integrin α11 not only promotes BMSCs osteogenic differentiation but also promotes chondrocyte proliferation. This result suggests that in chondrocytes, Clec11a may also bind to integrin α11 to promote the activation of Wnt signaling. The upstream–downstream relationship among Wnt signaling, Clec11a, and its receptor integrin α11 should be further explored to identify additional roles of Clec11a in skeletal system diseases.

**Figure 4:**
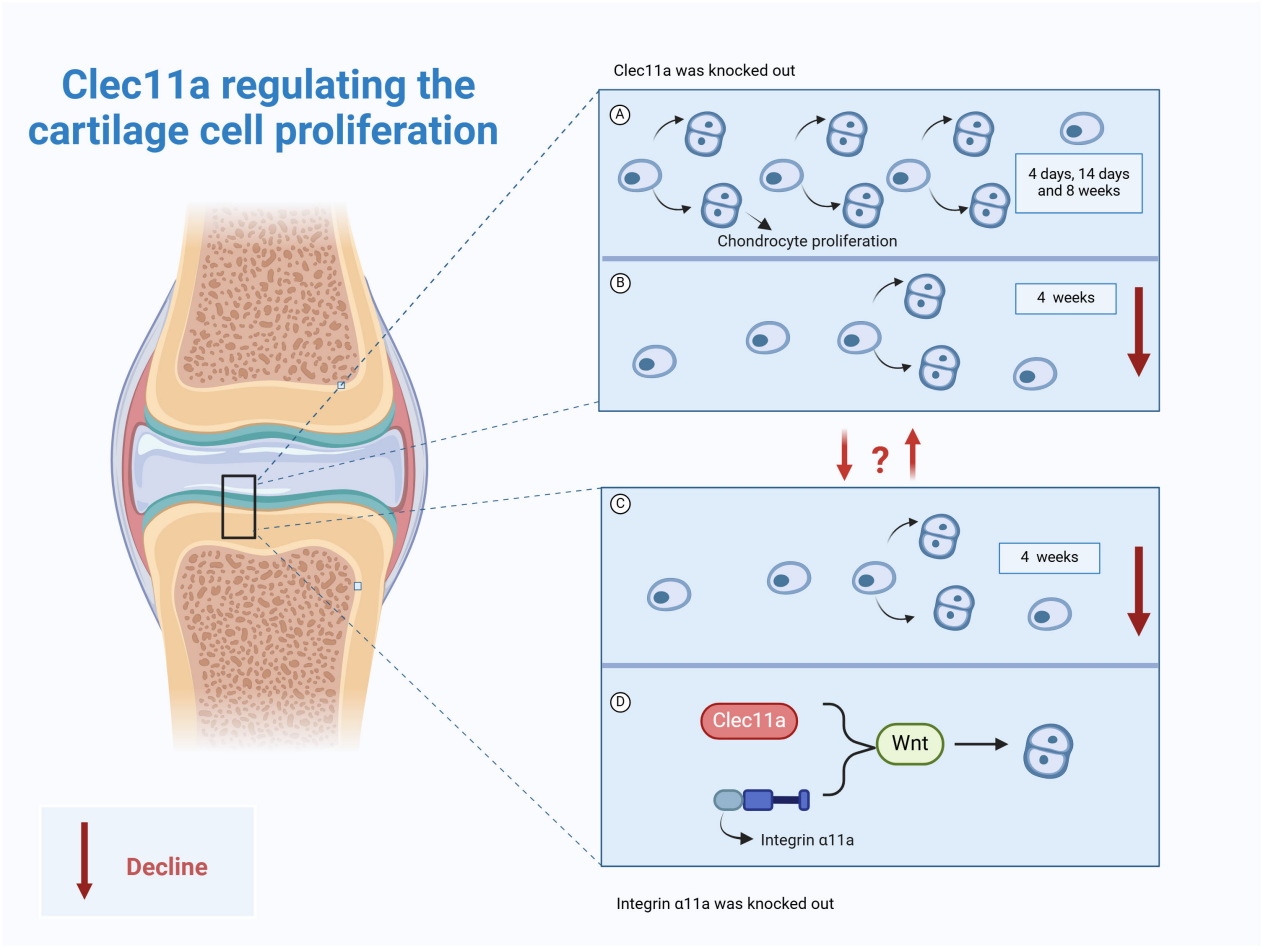
Role of Clec11a in chondrocyte proliferation. **(A)** Clec11a knockout had no effect on chondrocyte proliferation in 4-day, 14-day, and 8-week-old mice. **(B)** Clec11a knockout results in decreased chondrocyte proliferation in 4-week-old mice. **(C)** Knockout of integrin α11a can lead to decreased chondrocyte proliferation in 4-week-old mice. **(D)** Both Clec11a and integrin α11a promote chondrocyte proliferation by activating the Wnt signaling pathway.

Clec11a may be a promoter of chondrocyte proliferation and longitudinal bone growth in juvenile mice (**Figure 4**). However, its effect on chondrocyte proliferation varies among different age groups. For example, it was reported that Clec11a does not work in mice at the ages of 4 and 14 days. However, at 4 weeks of age, Clec11a promotes bone elongation by increasing growth plate chondrocyte proliferation, thereby increasing body length. The specific reasons for this phenomenon need to be further explored to provide a direction for the future use of Clec11a in the treatment of skeletal diseases in different age classes. In addition, integrin α11 has a high affinity for Clec11a and exhibits highly similar effects in cartilage. The direct interrelationship between the two should be investigated in the future to provide emerging drug targets for the treatment of skeletal diseases while developing a deeper understanding of the exact mechanisms underlying the action of Clec11a.

### 3.3 Potential roles for Clec11a in osteoblasts and osteoclasts

**Figure 5:**
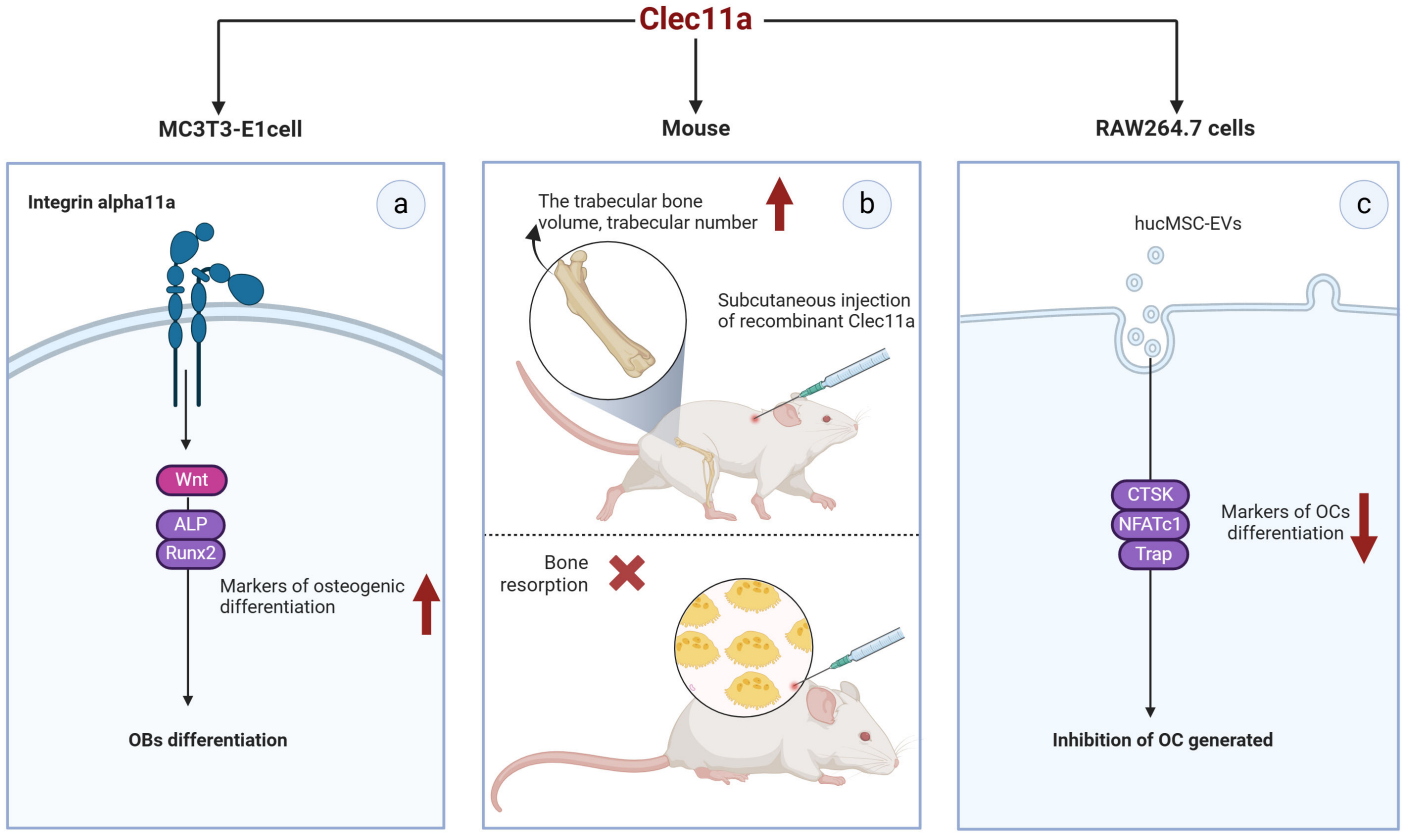
Potential of Clec11a in osteoblasts and osteoclasts. **(A)** Clec11a can bind to integrin α11 and respond to Wnt signaling to promote the expression of osteogenic markers ALP and Runx2, and promote the osteogenic differentiation of OB. **(B)** Recombinant Clec11a can increase the volume and number of trabecular bones at the metaphyseal end of the distal femur in mice, but does not affect bone resorption. **(C)** Clec11a can affect the osteoclast activity of hucMSC-EVS, reduce the expression of osteoclast markers CTSK, NFATc1 and Trap, and inhibit OC production.

Coupling of OBs and OCs is essential for maintaining bone construction and remodeling. The relationship of Clec11a with OBs and OCs has been less reported but still shows great promise in bone metabolic process (**Figure 5**). Clec11a levels in bone fragments containing OBs and osteocytes were found to be 80 times higher than those in the whole bone marrow or skeletal muscle cells (52).

MC3T3-E1MC3T3-E1 is a widely used mouse pre-osteoblast line that can mimic osteoblast differentiation to a certain extent. The transient expression of Clec11a in MC3T3-E1 cells promotes their differentiation toward OB in culture (7). Recombinant Clec11a treatment of MC3T3-E1 cells also significantly increased the osteogenic differentiation of these cells and activation of the Wnt signaling pathway (38). In contrast, Wnt target gene levels were significantly reduced in MC3T3-E1 cells with Clec11a deletion (38). Notably, the induction of OB differentiation by clec11a seems to be dependent on Itga11 as recombinant clec11a failed to induce osteogenesis in MC3T3-E1 cells where Itga11 was knocked down. Mechanistically speaking, Clec11a can selectively bind to integrin α11 and promote the osteogenic differentiation of OB in response to Wnt signals (38). This result suggests that Clec11a may play a role in OB osteogenic differentiation and could be a positive regulator of this process. Unfortunately, the presence of Clec11a in primary OB has not been reported at present, and the osteogenic differentiation potential of Clec11a should be verified in subsequent studies.

Notably, the role of Clec11a in OC remains controversial. For example, Yue et al. administered subcutaneous injections of recombinant Clec11a to 2-month-old wild-type mice for 28 days; they found that recombinant Clec11a dose-dependently increased the volume and number of trabeculae in the distal femoral epiphysis but did not affect bone resorption (7). In contrast, the number of OCs was significantly reduced after tail vein injection of hucMSC-EVs into 8-week-old wild-type mice (8). *In vitro*, RAW264.7 cells were induced to differentiate into OCs, and the quantification results after Trap staining revealed that hucMSC-EVS significantly inhibited the formation of mature OCs. However, silencing Clec11a in hucMSC-EVS could significantly improve the osteoclastogenic ability of hucMSC-EVS. Specifically, silencing Clec11a could obviously reverse the downregulation of genes such as NFATc1, Trap, and CTSK, which are the differentiation markers of OC induced by EVS (8). These data suggest that Clec11a is required for the inhibition of OC formation by hucMSC-EVs, which contradicts the *in vivo* results reported by Yue et al. ShRNA interference with Clec11a in hucMSCs may affect the expression of other genes, or some unknown mechanism may alter the molecular components in hucMSC-EVs and thus alter EV regulation. However, further studies are required to clarify these conflicting results. In addition, none of the primary bone marrow mononuclear macrophages (BMMs) from mice have been extracted in the reported study, which may be a research direction that needs to be further explored in the future.

It is known that Clec11a stimulates canonical Wnt signaling in BMSCs, chondrocytes, and MC3T3-E1 cells, thereby affecting bone construction and remodeling. Notably, the Wnt pathway regulates OB and OC communication through β-catenin-dependent (typical) and non-dependent (atypical) pathways (53).The non-canonical Wnt ligand Wnt5a is highly expressed in osteoblast lineage cells, which can induce OC production by activating the JNK MAPK pathway to regulate the expression of RANK in OC (54). Similarly, Wnt16 is also an OB-derived factor, and in addition to directly inhibiting OC production through the JNK MAPK pathway, Wnt16-induced JUN phosphorylation also upregulates OPG expression in OB, providing a direct mechanism for inhibiting OC production (55). Clec11a is known to activate the JNK signaling pathway in dental pulp cells and can mediate Wnt signaling to regulate bone construction and remodeling, suggesting that Clec11a may be an emerging cytokine linking OB and OC communication. However, our limited understanding of Clec11a requires more research to clarify the details, and exploring the close connection and mechanism between Clec11a and Wnt signals, as well as OB and OC crosstalk, maybe a potential direction for future research.

In summary, Clec11a may have extremely high osteogenic activity, which not only promotes osteogenic differentiation of BMSCs but also has great potential for regulating OB osteogenic activity. In terms of regulating OC, although there are some controversies, it is undeniable that it may affect the osteoclastic activity of hucMSC-EVS in some specific cases (**Table 1**). In the future, primary OBs and BMMs can be used to support the current research results. It is necessary to carry out multi-faceted basic experiments to further explore the coupling effect of Clec11a in OB and OC so as to provide more thinking and direction for the role of Clec11a in bone cells.

**Table 1:** Molecular mechanisms of Clec11a regulation of the skeletal system.

Bone cells	phenotype	Possible mechanisms	bibliography
BMSC	Promoting BMSCs osteogenic differentiation	hucMSC-EVs/Clec11a	(8)
		Clec11a/Fap	(45)
		Clec11a/integrin α11/Wnt	(38)
		Clec11a/PTH/Wnt	(63)
chondrocyte	Promote chondrocyte proliferation	Clec11a/Wnt	(9)
		Clec11a/Fap	(56)
OB	Promoting OB differentiation	Clec11a/integrin α11/Wnt	(38)
OC	Inhibition of mature OC formation	hucMSC-EVs/Clec11a	(8)

## 4 Role of Clec11a in bone diseases

### 4.1 Osteoarthritis

Osteoarthritis (OA) is the most common and highly prevalent chronic degenerative joint disease worldwide. Studies have estimated that more than 500 million people currently suffer from OA, which is on the verge of becoming the fourth leading cause of disability globally (57). A recent study by Fan et al. found that Fap not only has osteogenic inhibitory effects but is also expressed in synovial fibroblasts and significantly elevated in the OA synovium, which is positively correlated with OA severity (56). Notably, Clec11a, an endogenous inhibitor of Fap, was significantly lower in damaged OA cartilage (56). It was found through immunohistochemical detection of mouse knee joint tissues that Clec11a was expressed in the superficial layers of articular cartilage and meniscus (7). Following medial meniscus surgery in Clec11a knockout and Clec11a and Fap double knockout mice, Clec11a knockout mice exhibited significantly increased cartilage erosion, subchondral bone thickening, and synovitis (56). Notably, these phenotypes were significantly improved in the double-knockout mice. Moreover, weekly intra-articular administration of a FAP-selective small-molecule inhibitor similarly improved OA progression in Clec11a knockout mice (56). These findings suggest that Clec11a may prevent or delay OA progression by inhibiting Fap expression, which has been supported by subsequent experiments. For example, a previous study administered weekly intra-articular injections of recombinant Clec11a to mice 3 days after medial meniscus surgery for 8 weeks and revealed that cartilage erosion, subchondral bone thickening, and synovitis were significantly reduced in the Clec11a-treated group (56), thus further emphasizing the importance of Clec11a in the treatment of OA.

### 4.2 Osteoporosis

OP, a prevalent metabolic bone disorder mainly marked by reduced bone density and enhanced bone fragility, readily gives rise to fractures, disability, and imposes a substantial medical and economic burden (58). Thus, developing methods of preventing and treating OP represents a pressing issue. Daily administration of recombinant Clec11a significantly increased the volume and number of bone trabeculae, decreased trabecular spacing, and significantly increased the number of trabecular-associated OBs in 2-month-old ovariectomized mice (7). A secondary OP model established by intraperitoneal injection of dexamethasone produced similar results, suggesting that Clec11a can prevent bone loss by promoting bone formation, thereby preventing OP. Furthermore, Clec11a can not only prevent OP but is also highly effective at reversing OP. The same effect was achieved after the administration of Clec11a for 4 week- to 2-month-old ovariectomized mice that developed an OP phenotype (after 4 additional weeks) (7). This suggests that Clec11a can reverse the loss of bone trabeculae after ovariectomy-induced onset of OP, which is clinically significant for the treatment and alleviation of OP.

Currently, the treatment approaches for metabolic bone diseases such as OP are rather limited and often rely on drugs that reduce OC activity, such as bisphosphonates and RANKL antagonists, yet they have certain side effects. Long-term use of bisphosphates may overly suppress OC activity, causing atypical fractures (59); RANK-related drugs may also lead to an excessive rebound of OC precursors after drug withdrawal, increasing the risk of fractures (60). In contrast, Clec11a holds more advantages. It not only focuses on inhibiting bone resorption but also emphasizes promoting bone formation, promising more stable and long-lasting therapeutic effects. Furthermore, Clec11a seems to play a specific role in the differentiation of BMSCs into OB without affecting other cell lines differentiated from BMSC (61). This unique target point suggests that it might reduce adverse reactions similar to those of existing drugs. Among the current multidrugs, parathyroid hormone (PTH) can effectively regulate bone mass by stimulating bone formation and bone resorption (62). However, a single drug may have limited efficacy, and improving the synergy of individual drugs is an extremely promising strategy to combat OP. Recent reports have shown that PTH treatment increased Clec11a levels in premenopausal idiopathic OP patients and 2-month-old mice (63). A 4-week subcutaneous injection of PTH in Clec11a knockout mice resulted in decreases in bone volume, cortical bone thickness, and serum procollagen type 1 N-terminal prepeptide compared with that in the controls (63). This suggests that Clec11a deletion reduces the efficacy of PTH treatment to a degree. Mechanistic studies have shown that Clec11a facilitates the activation of the Wnt pathway by PTH to promote the osteogenic differentiation of BMSCs for the treatment of OP, which again demonstrates the possibility of using Clec11a for the treatment of OP. More importantly, the combination of PTH and Clec11a exerted better therapeutic effects than the single agents alone, showed a more pronounced increase in trabecular bone volume, trabecular number, and trabecular junction density, and exhibited a significant reduction in trabecular spacing (63). This suggests that recombinant PTH and Clec11a have cumulative effects on trabecular bone parameters, thus revealing new possibilities for the treatment of OP. The interaction between Clec11a and PTH should be further explored to test the safety of Clec11a in the treatment of OP, as well as the feasibility of using both in conjunction. In addition, pharmacological inhibition of Fap also attenuated OP induced in ovariectomized mice (63). As previously described, Clec11a inhibits Fap, thereby promoting vertebral mineralization during zebrafish development.

In summary, Clec11a has great potential value in the prevention and treatment of OP because it prevents the progression of OP in ovariectomized mice and plays a significant role in alleviating OP. From a clinical perspective, these findings reveal that Clec11a has extremely broad potential therapeutic value. The interrelationships between Clec11a and bisphosphonates, PTH, Fap, and other factors should be further explored to provide a more effective and multi-selective therapeutic regimen for the treatment of OP.

### 4.3 Other bone-related diseases

Osteosarcoma is a common malignant bone tumor, and its overall survival rate has stagnated at 60–70% over the past 60 years (64, 65). Thus, the development of more efficient therapeutic approaches to improve the survival rate of osteosarcoma is of great significance. By performing scRNA-Seq on advanced osteosarcoma tissues, a novel Clec11a B cell subpopulation with high expression of Clec11a, MMP-9, and bone-bridging proteins was identified, suggesting that Clec11a B cells may be involved in bone metabolism (66). Subsequent enrichment analysis showed that these highly expressed genes were associated with extracellular matrix-receptor interactions, protein digestion and absorption, mineral uptake, and estrogen signaling pathways (66), further suggesting that they may be involved in bone metabolism, osteoblastoma cell renewal, proliferation, and invasion. These findings imply that Clec11a B cells may influence osteosarcoma progression by regulating tumor cell or T-cell subcluster activity. Thus, Clec11a B cells may represent an emerging target for osteosarcoma treatment.

Multiple myeloma is an incurable plasma cell malignancy of the bone marrow, with up to 30,000 new cases being annually reported in the United States alone (67, 68). Molecular and clinical data from 450 patients with multiple myeloma measured using a network biology approach confirmed that Clec11a serves as a novel modulator and candidate target for the treatment of myeloma (69). Type I neurofibroma is a tumor susceptibility syndrome caused by mutations in the heterozygous NF1 gene (70) and represents a key and difficult area of current medical diagnosis and treatment. The onset of the disease is often accompanied by multifunctional somatic secondary manifestations, including complications such as bone pseudoarthrosis after fracture (71). A recent study showed that impaired Clec11a-Itga11-Wnt signaling is involved in the pathogenesis of NF1-associated skeletal diseases (72), which not only provides new insights into the molecular pathogenesis of NF1-associated pseudoarthrosis but also reinforces the potential usefulness of Clec11a in bone tumors.

In conclusion, Clec11a may have great potential in the treatment of bone diseases, including chronic bone diseases, such as OA and OP, as well as osteosarcoma, multiple myeloma, and other diseases. In the future, the mechanisms underlying its ability to regulate bone diseases, as well as its possible contraindications and dosages for the clinical treatment of bone diseases, should be further explored to provide new possibilities for the treatment of bone diseases.

## 5 Effect of exercise on Clec11a expression

Exercise provides many benefits to the skeletal system, acts directly or indirectly on almost all bone cell types, and affects many aspects of bone construction and remodeling, such as promoting bone formation by stimulating the osteogenic differentiation of BMSCs and the activity of OBs and osteocytes (73, 74). Exercise is extremely relevant to Piezo1, which is a mechanosensitive cation channel that senses a variety of mechanical stimuli, including membrane stretch and fluid shear stress, and translates them into biochemical stimuli, thereby amplifying biological actions within the membrane (75, 76). Based on its structural and functional uniqueness, Piezo1 regulates BMSCs, OBs, OCs, and adipocytes, plays a role in the maintenance of bone homeostasis (4, 77), and is critical for maintaining bone homeostasis.

LepR-positive cells, including BMSCs, OBs, and adipose progenitor cells (75, 78–82) expressing LepR-positive BMSCs in the adult bone marrow is an important source of growth factors (83, 84); however, only a few markers that can be used to distinguish and compare their functions have been identified. Notably, a transgenic mouse model using a Clec11a-driven fluorescent reporter gene to label osteoblast lineages showed that LepR and Clec11a could define different cell subsets with double-positive cells of LepR and Clec11a around small arteries tending to differentiate osteoblastically and LepR-positive and Clec11a-negative cells around the medullary sinus tending to differentiate into adipocytes (10). Bone marrow is sensitive to mechanical stimuli (85), and in-depth studies have shown that small arteries of the bone marrow are better able to sense physical and mechanical forces than the medullary sinus. Moreover, 4 weeks of running wheel exercise not only increased bone mass in 18-month-old Clec11a-positive mice but also increased the number of Clec11a-positive cells around the small arteries (10). Conversely, suspension of the hind limbs of 2-month-old Clec11a-positive mice for only 2 weeks resulted in a significant decrease in bone mineral density, cortical bone thickness, and Clec11a-positive cell number in the femurs of hind limbs. Mechanistic studies have shown that mechanical stimulation of Clec11a-positive cells activated Piezo1 signaling, while Clec11a-positive cell-specific knockdown of Piezo1 significantly reduced bone density and cortical bone thickness in mice (10), suggesting that Clec11a-positive cells promote osteogenesis by sensing mechanical stress through the Piezo1 signaling pathway. This not only reveals a new mechanism by which exercise improves bone quality but also suggests a potential relationship between Clec11a and exercise, thus providing stronger evidence for the feasibility of Clec11a in the treatment of bone diseases.

Taken together, Clec11a may promote osteogenesis by responding to mechanical stimuli through the mechanosensitive ion channel Piezo1. Notably, Piezo1 expression decreases with aging (86), which may lead to a decrease in the effect of Clec11a on bone. As mentioned previously, integrins respond to mechanical stimuli; however, whether Clec11a binds its receptor integrin α11 in response to mechanical stimuli has not been determined. The connection between Clec11a, bone, and movement, along with the underlying molecular mechanisms, should be further explored to provide new treatments for bone diseases.

## 6 Summary and outlook

To date, a large number of studies have focused on unraveling the pathogenesis of bone diseases, such as OP and OA, and emerging therapeutic approaches. Clec11a is a recently identified factor that exerts a positive effect on bone construction and remodeling; however, the molecular mechanisms underlying its ability to regulate the skeletal system have not been extensively investigated. Studies on the effects of Clec11a on bone cells have focused on BMSCs and chondrocytes. Clec11a has high osteogenic activity and promotes the differentiation of BMSCs toward osteogenesis, and it can also promote chondrocyte proliferation to achieve longitudinal bone growth. Although Clec11a represents an emerging highly osteogenic active factor, its effects on OB and OC remain poorly understood and need to be further explored in large-scale and focused basic studies in the future. More importantly, Clec11a has great pharmacological potential in the treatment of bone diseases because it can regulate a variety of bone diseases and be combined with PTH to produce a “1 + 1>2” therapeutic effect on OP. The relationship between Clec11a and locomotion was not fully investigated in the current study; however, Clec11a has been shown to respond to mechanical stimulation through Piezo1 to produce beneficial effects on bones. In the future, we will further explore the wider connection between Clec11a and mechanical stimulation, which may lead to a major breakthrough in its connection with bone biology. In conclusion, utilizing Clec11a and its upstream and downstream factors for the development of new drugs and clinical treatment and exploring the molecular mechanisms of Clec11a with respect to movement and bone represent promising research directions. Finally, in addition to its inherent characteristics, there may also be numerous external factors that influence the role of Clec11a on bone health, such as drugs, nutrition, and light exposure, which represents another avenue of future research. These investigations are expected to provide new insights into many areas of basic science, enhancing our understanding of the mechanisms underlying numerous skeletal disorders and facilitating the development of more efficient therapeutic options for the clinical treatment of bone diseases.
